# Members of Bitter Taste Receptor Cluster *Tas2r143/Tas2r135/Tas2r126* Are Expressed in the Epithelium of Murine Airways and Other Non-gustatory Tissues

**DOI:** 10.3389/fphys.2017.00849

**Published:** 2017-10-30

**Authors:** Shuya Liu, Shun Lu, Rui Xu, Ann Atzberger, Stefan Günther, Nina Wettschureck, Stefan Offermanns

**Affiliations:** ^1^Department of Pharmacology, Max Planck Institute for Heart and Lung Research, Bad Nauheim, Germany; ^2^Flow Cytometry Service Facility, Max Planck Institute for Heart and Lung Research, Bad Nauheim, Germany; ^3^ECCPS Bioinformatics and Deep Sequencing Platform, Max Planck Institute for Heart and Lung Research, Bad Nauheim, Germany; ^4^Medical Faculty, Goethe University Frankfurt, Frankfurt, Germany

**Keywords:** Tas2r143, Tas2r135, Tas2r126, chemosensory cells, tuft cells

## Abstract

The mouse bitter taste receptors Tas2r143, Tas2r135, and Tas2r126 are encoded by genes that cluster on chromosome 6 and have been suggested to be expressed under common regulatory elements. Previous studies indicated that the *Tas2r143/Tas2r135/Tas2r126* cluster is expressed in the heart, but other organs had not been systematically analyzed. In order to investigate the expression of this bitter taste receptor gene cluster in non-gustatory tissues, we generated a BAC (bacterial artificial chromosome) based transgenic mouse line, expressing CreERT2 under the control of the *Tas2r143* promoter. After crossing this line with a mouse line expressing EGFP after Cre-mediated recombination, we were able to validate the *Tas2r143*-CreERT2 transgenic mouse line and monitor the expression of *Tas2r143*. EGFP-positive cells, indicating expression of members of the cluster, were found in about 47% of taste buds, and could also be found in several other organs. A population of EGFP-positive cells was identified in thymic epithelial cells, in the lamina propria of the intestine and in vascular smooth muscle cells of cardiac blood vessels. EGFP-positive cells were also identified in the epithelium of organs readily exposed to pathogens including lower airways, the gastrointestinal tract, urethra, vagina, and cervix. With respect to the function of cells expressing this bitter taste receptor cluster, RNA-seq analysis in EGFP-positive cells isolated from the epithelium of trachea and stomach showed expression of genes related to innate immunity. These data further support the concept that bitter taste receptors serve functions outside the gustatory system.

## Introduction

Mammals sense a wide variety of compounds as bitter to prevent the ingestion of toxic substances. Bitter taste receptors are type 2 taste receptors (T2Rs) and belong to the superfamily of G-protein-coupled receptors (GPCRs). T2Rs are expressed primarily by type II taste receptor cells in taste buds of the tongue and share with sweet/umami taste receptors key molecules in their downstream signaling cascade. The canonical signal transduction involves activation of the G-protein gustducin, phospholipase C (PLC) ß2 and production of inositol triphosphate (IP3). IP3 induces an intracellular Ca^2+^ increase, which activates transient receptor potential channel M5 (TRPM5), leading to cation influx, membrane depolarization and release of neurotransmitters such as ATP and acetylcholine (Chandrashekar et al., 2006; Roper, 2013). Unlike sweet/umami taste receptors, bitter taste receptors are capable of detecting diverse chemical compounds, and individual bitter taste receptors show different degrees of ligand selectivity (Lossow et al., 2016).

Recent studies showed expression of bitter taste receptors and key molecules of the downstream taste signaling cascade outside the gustatory system and suggested important physiological functions of bitter taste receptors in various other organs (Finger and Kinnamon, 2011; Pydi et al., 2014; Lu et al., 2017). Reporter mice indicating expression of TRPM5 demonstrated the presence of solitary chemosensory cells (referred to as brush cells or tuft cells) in organs and tissues such as the airway, gastrointestinal tract and urethra (Bezencon et al., 2007; Tizzano et al., 2010; Kusumakshi et al., 2015). It has been suggested that such solitary chemosensory cells detect bacterial quorum-sensing molecules in the murine airway (Tizzano et al., 2010; Krasteva et al., 2012; Saunders et al., 2014), and initiate type 2 immune responses to helminth infections in the murine intestine (Gerbe et al., 2016; Howitt et al., 2016; von Moltke et al., 2016). Importantly, *TAS2R38* polymorphisms were shown to be linked to susceptibility to upper respiratory infection in human (Lee et al., 2012). These studies indicate that bitter taste receptors may be activated by particular pathogens and be involved in distinct physiological processes. Therefore, analysis of individual bitter taste receptor expression can help to elucidate their functions outside the gustatory system. However, expression of individual bitter taste receptors has been less investigated. Transgenic mice indicating expression of *Tas2r131* and *Tas2r105* have shown that these receptors are expressed in the thymus, trachea, ovary, and testis as well as in the kidney, small intestine, and testis, respectively (Li and Zhou, 2012; Voigt et al., 2012, 2015; Gu et al., 2015; Liu et al., 2015; Soultanova et al., 2015).

There are 25 and 35 functional bitter taste receptors in human and in mice, respectively. The human T2R genes cluster on chromosomes 5, 7, and 12 and the murine T2R genes cluster on chromosomes 2, 6, and 15 (Go et al., 2005). Real-time qPCR showed that the bitter taste receptor cluster *Tas2r143*/*Tas2r135*/*Tas2r126* is expressed in rodent hearts and upregulated under starvation, indicating that these genes could be regulated under a common element (Foster et al., 2013). *In silico* analysis based on datasets from the ENCODE (Encyclopedia of DNA Elements) Consortium showed overlapping histone marks and DNase I hypersensitive sites upstream of *Tas2r143*, indicating that *Tas2r143, Tas2r135*, and *Tas2r126* share common *cis*-regulatory regions (Foster et al., 2015). In addition, Tas2r143 and Tas2r135 of this bitter taste receptor cluster have been detected by real-time qPCR in murine vascular smooth cells (Lund et al., 2013) and in high-fat-diet induced mouse fat pads (Avau et al., 2015), respectively.

In order to investigate the expression of the bitter taste receptor cluster *Tas2r143/Tas2r135/Tas2r126* in non-gustatory tissues in more detail, we generated a BAC (bacterial artificial chromosome) based *Tas2r143*-CreERT2 transgenic mouse line. By crossing *Tas2r143*-CreERT2 with Rosa26^flox−mT−stop−flox−mG^ Cre-reporter mice we were able to validate this transgenic mouse line, monitor the expression of EGFP, and characterize the expression pattern of this bitter taste receptor cluster.

## Materials and methods

### Animals

To generate *Tas2r143*-CreERT2 transgenic mice expressing CreERT2 under the control of the *Tas2r143* promoter, the BAC clone RP23-316O11 (CHORI, CA, USA) from mouse chromosome 6 was used, containing the *Tas2r143, Tas2r135*, and *Tas2r126* genes. The genomic sequence between the start codon of *Tas2r143* and the stop codon of *Tas2r126* (35.2 kb) on the BAC was replaced by a cassette carrying the CreERT2 cDNA followed by a polyadenylation (pA) signal and a FRT-flanked ampicillin resistance cassette using Red/ET recombination kit (Gene Bridges). Correct targeting was verified by restriction digestion (BaeI, HpaI, and SwaI) and DNA sequencing. After Flp-mediated excision of the ampicillin resistance cassette and linearization (NotI), the recombined BAC was injected into C57BL/6 oocytes. Targeted offspring was genotyped for BAC insertion by genomic PCR. Following primers were used: P1 (forward): 5′-CAG GAG TCA TTG AAC TGG GAG-3′; P2 (reverse): 5′-CAG CAT CCA CAT TCT CCT TTC TGA-3′; P3 (forward): 5′-GCA TCG CAT TGT CTG AGT AGG T-3′; P4 (reverse): 5′-CAG ACA TGA AAG GAA CAG GAC AT-3′; PCR with P1/P2 and P3/P4 amplified a 614 bp and a 418 bp fragment, respectively, representing the correctly inserted CreERT2. Three independently generated *Tas2r143*-CreERT2 lines were crossed with the Cre-reporter line Rosa26^flox−mT−stop−flox−mG^ (Jackson Lab, Stock 007576) and analyzed, showing similar expression pattern. Nuclear translocation of the CreERT2 fusion protein was induced in animals by intraperitoneal injection of 1 mg tamoxifen (Sigma T5648) per day for 5 consecutive days. Five days after the last tamoxifen injection the animals were analyzed. In the absence of tamoxifen treatment, all *Tas2r143*-CreERT2 lines did not show any basal recombination. We therefore in some cases used the Rosa26^flox−mT−stop−flox−mG^ mouse line as a control.

Animals were kept on a C57BL/6 background and housed under a 12 h light-dark cycle with free access to food and water and under pathogen-free conditions. The Animal Ethics Committee of the Regierungspräsidium Darmstadt approved all animal procedures (Protocol No. B2/1016, B2/1112).

### Immunofluorescence microscopy

Tissues and organs were fixed in 4% PFA in PBS at 4°C for 12 h, subsequently incubated in 30% sucrose overnight for cryopreservation and embedded in O.C.T. tissue freezing medium (Sakura®, The Netherlands) and stored at −80°C before sectioning. For immunofluorescence analysis, cryosections (8–10 μm) were fixed in 4% PFA for 5 min or in acetone at −20°C for 10 min, washed three times in PBS, blocked with 5% BSA + 0.1% Triton X-100 in PBS for 30 min at room temperature. The blocked sections were incubated overnight at 4°C with following primary antibodies: rabbit anti-DCLK1 (ab31704, Abcam), goat anti-ChAT (AB144P, Millipore), goat anti-Gα gust (I-20; sc-395, Santa Cruz), rabbit anti-PLCß2 (sc-206, Santa Cruz), rat anti-CD31 (550274, BD Bioscience), rabbit anti-K5 (ab53121, Abcam), rabbit anti-K10 (905401, BioLegend), rabbit anti-K18 (clone SP69, M3690, Spring Bio), biotin anti-αSMA (ab125057, Abcam), Alexa Fluor® 647 anti-mouse CD3 (100209, BioLegend). After primary antibody incubation, sections were washed three times in PBS and incubated in the dark for 1 h at room temperature with appropriate secondary antibodies: goat anti-rabbit IgG Alexa Fluor® 647 (A32733), donkey anti-goat IgG Alexa Fluor® 647 (A21447), goat anti-rat lgG Cy5® (A10525), and streptavidin Alexa Fluor® 647 (S21374) from Life Technologies. Cell nuclei were labeled with DAPI (D3571, Invitrogen). After washing three times in PBS, sections were mounted and images were taken by SP5 confocal microscope (Leica). DAPI was detected at a wavelength of 405 nm, EGFP at 488 nm, Tomato at 561 nm and Alexa Fluor® 647 and Cy5® at 633 nm. Images were analyzed by Leica software (LAS AF Lite).

The Rosa26^flox−mT−stop−flox−mG^ mouse line expresses either EGFP or Tomato in a mutually exclusive fashion, and both fluorescent proteins are present in the cell in a membrane-associated form (Muzumdar et al., 2007). Therefore, in confocal images with a relatively large slice thickness, the overlay of the fluorescent images obtained at 488 nm and 561 nm in some cases wrongly suggests colocalization. Colocalization was only concluded when the signal from EGFP colocalized with an immunohistochemical signal obtained at 633 nm in an area which was not restricted to the direct cell-cell contact site.

### Quantitative assessment of EGFP-positive cells

Tongues (*n* = 4) were sectioned longitudinally and 10–15 cryosections were analyzed for each mouse. Taste buds were identified based on their onion-like morphology visualized by Tomato. Number of total and EGFP-positive taste buds was counted manually throughout the section using the 20x magnification field. Hearts (*n* = 5) were sectioned longitudinally and 12–15 independent images were taken at 20x magnification per mouse. Muscularized vessels were identified by αSMA. Number of total and EGFP-positive muscularized vessels was counted manually. Tracheas (*n* = 4) between the larynx and the bifurcation were dissected and sectioned longitudinally. Twenty to twenty-four independent images were taken at 20x magnification for each mouse. Cell counting was performed on the basis of nuclear staining with DAPI and cell markers. EGFP-positive cells, DCLK1-positive tuft cells, and total epithelial cells were counted manually. Stomachs (*n* = 4) were opened along the greater curvature, embedded flatly and sectioned as reported previously (Eberle et al., 2013). Images were taken at 20x magnification and gastric units, which could be viewed longitudinally, were analyzed. EGFP-positive cells, DCLK1-positive tuft cells and total epithelial cells were counted manually in 110–150 longitudinal gastric units for each mouse. Female urethra (*n* = 3) were sectioned longitudinally and 20–22 independent images were taken at 20x magnification for each mouse. EGFP-positive cells were counted manually and epithelial cells were counted by ImageJ (NIH) based on nuclear staining with DAPI in the epithelial layer.

### Isolation of mouse tongue epithelium

The isolation protocol was reported previously (Voigt et al., 2012). Briefly, the tongue was removed and an enzyme mix containing 2.5 mg/ml dispase II (D4693, Sigma), 1 mg/ml collagenase A (10103578001, Roche) and 0.5 mg/ml DNase I (A3778,0100, AppliChem) in PBS was injected under the epithelial layer. The injected tongue was incubated for 15 min at room temperature and the epithelium was peeled, washed in PBS and prepared for RNA isolation.

### Isolation of mouse tracheal cells

The isolation protocol was reported previously (Rock et al., 2009). Briefly, tracheas were cut into pieces, incubated in 16 U/ml dispase (17105-041, Gibco) in PBS for 30 min at room temperature and centrifuged at 350 g for 5 min. The digested tracheas were resuspended in 0.1% trypsin (25200056, Gibco), 1.6 mM EDTA in PBS and incubated for 20 min at 37°C. Trypsin was inactivated by adding DMEM with 5% FBS. The cell suspension was passed through a 40-μm cell strainer (431750, Corning). The filtered cells were centrifuged at 350 g for 5 min and the cell pellet was resuspended in DMEM with 5% FBS for FACS analysis.

### Isolation of mouse gastric mucosal cells

The isolation protocol was reported previously (Sakata et al., 2012). Briefly, stomachs were inverted, inflated with ~1 ml PBS, and digested with 0.05 mg/ml Liberase TM (05401119001, Roche) in DMEM while being slowly shaken at 37°C for 90 min. The cell suspension was centrifuged at 350 g for 5 min and the cell pellet was resuspended in 0.25% trypsin–EDTA for 5 min at 37°C. Trypsin was inactivated by adding DMEM with 10% FBS. The cell suspension was passed through a 100-μm cell strainer (431752, Corning). The filtered cells were centrifuged at 350 g for 5 min and the cell pellet was resuspended in DMEM with 10% FBS for FACS analysis.

### Fluorescence-activated cell sorting (FACs)

Cells isolated from reporter mice were stained with EpCAM Alexa Fluor® 647 (118211, BioLegend) to label epithelial cells. Cells isolated from Rosa26^flox−mT−stop−flox−mG^ mice were not stained and served as EGFP/EpCAM-negative control. Single live cells were detected by gating on SSC/FSC and selecting DAPI-negative cells. EGFP-positive cells were identified and sorted based on EGFP-positive/DAPI-negative expression. Tomato-positive cells were identified and sorted based on Tomato-positive/EpCAM-positive/EGFP-negative/DAPI-negative expression. Same number of EGFP- and Tomato-positive cells was isolated from the tamoxifen-treated reporter mice and sorted directly in lysis buffer for RNA isolation.

### Real-time qPCR

RNA was prepared using RNAeasy Kit (74004, Qiagen) and treated with TURBO™ DNase (AM1907, Ambion) to remove genomic DNA. First-strand cDNA was synthesized using ProtoScript II Reverse Transcriptase (M0368, New England BioLabs). Real-time qPCR primers were designed with the online tool provided by Roche, and quantification was performed using the LightCycler 480 Probe Master System (Roche). Genomic DNA was used as a universal standard to calculate gene copy number per ng of RNA. Relative expression levels were obtained by normalization to *Actb* mRNA. Following real-time qPCR primers were used: *Tas2r143* (forward): 5′-CAG GCA TCT TTT TGA ACT CCA-3′; *Tas2r143* (reverse): 5′-TCT TCA GGG CCT TTC TCA GT-3′; *Tas2r135* (forward): 5′-CCA TCA TGT CCA CAG GAG AA-3′; *Tas2r135* (reverse): 5′-TCA GTA GTC TGA CAT CCA AGA ACT GT-3′; *Tas2r126* (forward): 5′-GTG TGT GGG ATT GGT CAA CA-3′; *Tas2r126* (reverse): 5′-GCT CCC GGA GTA CTC AAC C-3′; *Actb* (forward): 5′-AAA TCG TGC GTG ACA TCA AA-3′; *Actb* (reverse): 5′-TCT CCA GGG AGG AAG AGG AT-3′.

### Nanostring analysis of bitter taste receptors expression in the adult mouse heart

The isolation protocol of adult mouse cardiomyocytes was reported previously (Takefuji et al., 2012). Briefly, the heart was removed quickly and cannulated from the aorta with a blunted 27G needle to allow retrograde perfusion of the coronary arteries. The heart was first washed with 50 ml perfusion buffer (113 mM NaCl, 4.7 mM KCl, 0.6 mM KH_2_PO_4_, 1.2 mM MgSO_4_, 12 mM NaHCO_3_, 10 mM KHCO_3_, 10 mM HEPES, 30 mM Taurine, 10 mM 2,3-Butanedione monoxime, 5.5 mM Glucose, pH 7.46), then digested with 75 ml digesting buffer (0.05 mg/ml Liberase DH (05401089001, Roche) and 12.5 μM CaCl_2_ in perfusion buffer). The heart was removed from the perfusion apparatus and minced with a forceps in digesting buffer. Undissociated clumps were removed by filtration through 100 μm nylon mesh. Cardiomyocytes were enriched by three times centrifugation at 50 g for 1 min. RNA was isolated by RNeasy Kit (Qiagen). NanoString analyses were performed as described previously (Khan et al., 2011). In brief, 500 ng RNA was applied in a total volume of 30 μl in the assay. Barcodes were counted for ~1,150 fields of view per sample. Counts were first normalized to the geometric mean of the positive control spike count, then a background correction was done by subtracting the mean + two standard deviations of the eight negative control counts for each lane. Values that were <20 were fixed to background level. Relative expression levels were obtained by normalization to reference gene *Gusb*.

### RNA-Seq analysis of EGFP-positive cells in reporter mice

EGFP- and Tomato-positive cells were sorted by FACS and pooled from three reporter mice of one of the transgenic lines. RNA was isolated from EGFP- and Tomato-positive cells using the RNeasy micro Kit (Qiagen) combined with on-column DNase digestion (DNase-Free DNase Set, Qiagen) to avoid contamination by genomic DNA. RNA and library preparation integrity were verified with a BioAnalyzer 2100 (Agilent) or LabChip Gx Touch 24 (Perkin Elmer). RNA amount was adjusted on number of isolated cells by FACS and ~250 ng of total RNA was used as input for SMART-Seq® v4 Ultra® Low Input RNA Kit (Takara Clontech) for cDNA pre-amplification. Obtained full length cDNA was checked on LabChip and fragmented by Ultrasonication by E220 machine (Covaris). Final Library Preparation was performed by Low Input Library Prep Kit v2 (Takara Clontech). Sequencing was performed on the NextSeq500 instrument (Illumina) using v2 chemistry, resulting in minimum of 28 M reads per library with 1 × 75 bp single end setup. The resulting raw reads were assessed for quality, adapter content and duplication rates with FastQC (Andrews, 2010). Reaper version 13–100 was employed to trim reads after a quality drop below a mean of Q20 in a window of 10 nucleotides (Davis et al., 2013). Only reads between 30 and 150 nucleotides were cleared for further analyses. Trimmed and filtered reads were aligned vs. the Ensembl mouse genome version mm10 (GRCm38) using STAR 2.4.0a with the parameter “—outFilterMismatchNoverLmax 0.1” to increase the maximum ratio of mismatches to mapped length to 10% (Dobin et al., 2013). The number of reads aligning to genes was counted with featureCounts 1.4.5-p1 tool from the Subread package (Liao et al., 2014). Only reads mapping at least partially inside exons were admitted and aggregated per gene. Reads overlapping multiple genes or aligning to multiple regions were excluded. The Ensemble annotation was enriched with UniProt data (release 06.06.2014) based on Ensembl gene identifiers (Activities at the Universal Protein Resource, UniProt). The relative expression of genes in EGFP-positive cells compared to Tomato-positive cells was calculated as log_2_(EGFP/Tomato).

## Results

### Generation of tamoxifen-inducible *Tas2r143*-reporter mice

**Figure 1:**
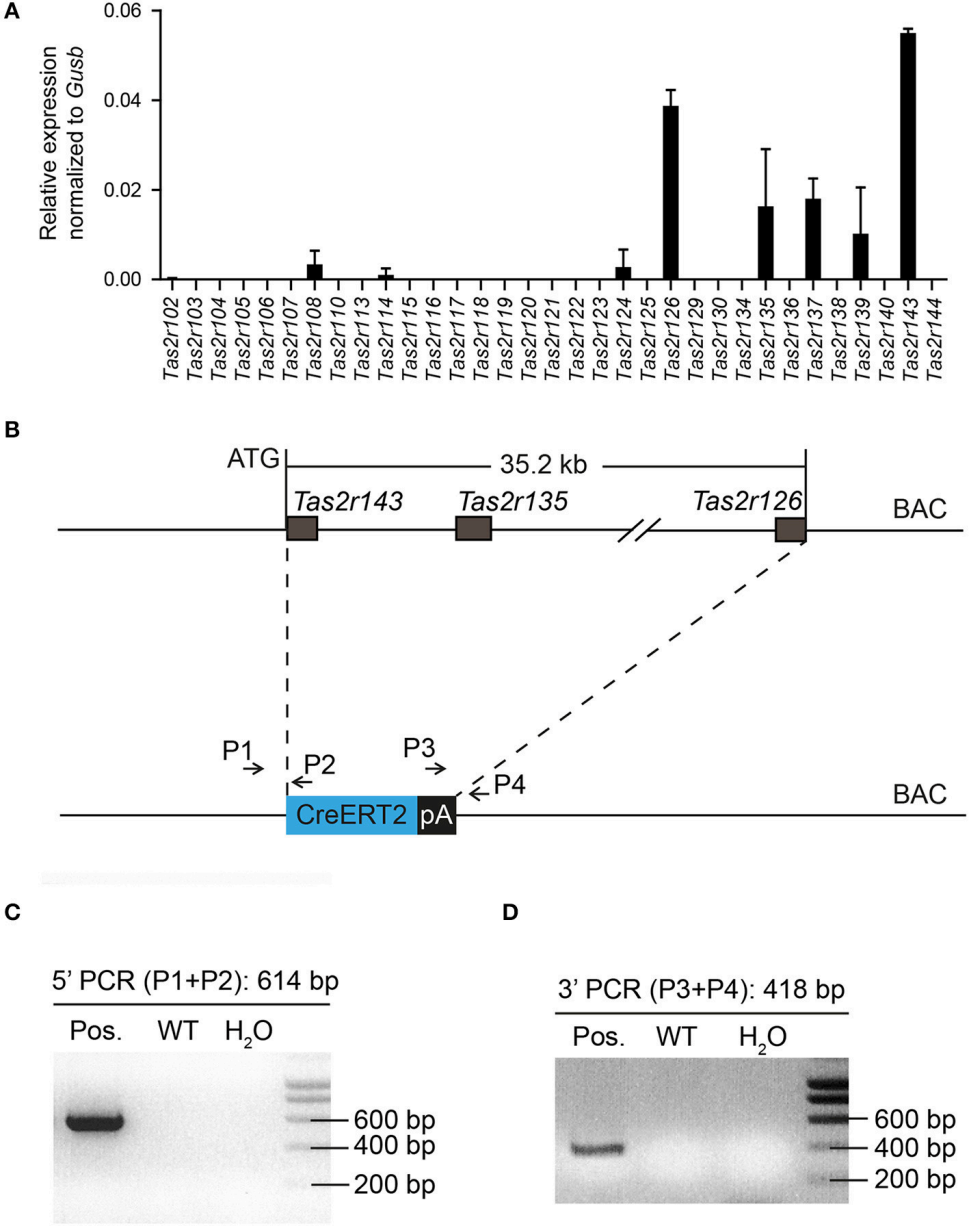
Generation of *Tas2r143*-CreERT2 transgenic mice. **(A)** NanoString analysis of mRNA expression levels for bitter taste receptors in the murine heart. (*n* = 2, error bars represent mean ± *SD*). **(B)** The genomic sequence between the start codon of *Tas2r143* and the stop codon of *Tas2r126* (35.2 kb) on BAC RP23-316O11 was replaced by a cassette carrying the CreERT2 cDNA followed by a polyadenylation (pA) signal. **(C,D)** Offspring of transgenic mice was genotyped by genomic PCR with primers P1/P2 and P3/P4 identifying a 614 bp and a 418 bp fragment, respectively, which indicate the correctly inserted CreERT2.

The murine bitter taste receptor genes *Tas2r143, Tas2r135*, and *Tas2r126* are located in close proximity to each other on chromosome 6 without any other known genes or coding sequences located within this area. NanoString analysis confirmed that these receptors were expressed in the heart (Figure 1A), suggesting that these three receptors may be transcribed under a common regulatory element (Foster et al., 2015). To analyze the expression of this cluster in mice we generated a BAC based *Tas2r143*-CreERT2 transgenic mouse line by inserting the cDNA encoding CreERT2 between the start codon of *Tas2r143* and the stop codon of *Tas2r126* (35.2 kb; Figure 1B). Transgenic mice were genotyped by genomic PCR to identify correct insertion of CreERT2 (Figures 1C,D).

**Figure 2:**
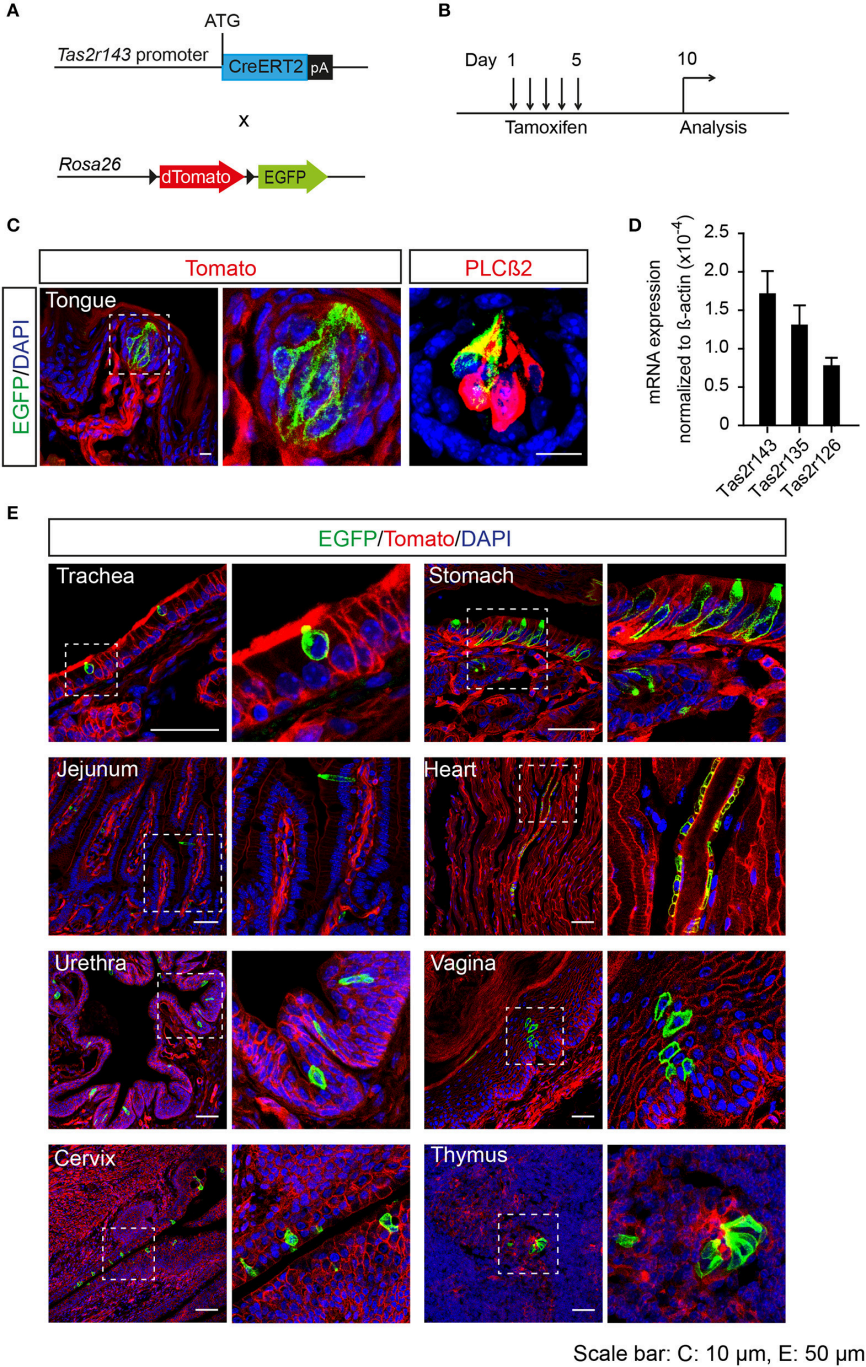
Generation of tamoxifen-inducible *Tas2r143*-reporter mice. **(A)** *Tas2r143*-CreERT2 mice were crossed with Cre-reporter mice (Rosa26^flox−mT−stop−flox−mG^) to generate *Tas2r143*-CreERT2; Rosa26^flox−mT−stop−flox−mG^ reporter mice. **(B)** Cre recombinase activity was induced by tamoxifen injection on 5 consecutive days. Reporter mice were analyzed 5 days after the last injection. **(C)** After tamoxifen injection, EGFP-positive cells were detected in taste receptor cells, stained by anti-PLCß2 antibody, in taste buds of the tongue. **(D)** *Tas2r143, Tas2r135*, and *Tas2r126* were detected in the tongue epithelium by real-time qPCR (*n* = 3, error bars represent mean ± *SD*). **(E)** EGFP-positive cells were detected in cryosections from various organs. Nuclei were counterstained with DAPI. Squares indicate enlarged areas. Scale bars: 10 μm **(C)**, 50 μm **(E)**.

To analyze the recombination function of *Tas2r143*-CreERT2 transgenic mice we crossed these mice with the Cre-reporter mouse line Rosa26^flox−mT−stop−flox−mG^ to generate *Tas2r143*-CreERT2;Rosa26^flox−mT−stop−flox−mG^ reporter mice (hereafter *Tas2r143*-reporter mice; Figure 2A). In this reporter strain the red fluorescent protein Tomato is ubiquitously expressed at baseline and switches to enhanced green fluorescent protein (EGFP) in all cells that have undergone Cre-mediated recombination. Activation of Cre recombinase was induced by tamoxifen application in adult mice for 5 days, and these mice were analyzed 5 days after the last injection (Figure 2B). EGFP-positive cells were detected in 47 ± 7% (*n* = 4, mean ± *SD*) of all taste buds in the tongue, and these cells were positive for the taste receptor cell marker PLCß2 (Roper, 2013; Figure 2C). We isolated the epithelium from the tongue and confirmed the expression of *Tas2r143, Tas2r135*, and *Tas2r126* by performing real-time qPCR (Figure 2D). Beyond the tongue, EGFP-positive cells were detected in the trachea, stomach, jejunum, urethra, vagina, cervix, and thymus, as well as in the heart (Figure 2E). To validate and characterize the EGFP-positive cells we took a closer look at various organs.

### Expression of EGFP-positive cells in the respiratory epithelium

**Figure 3:**
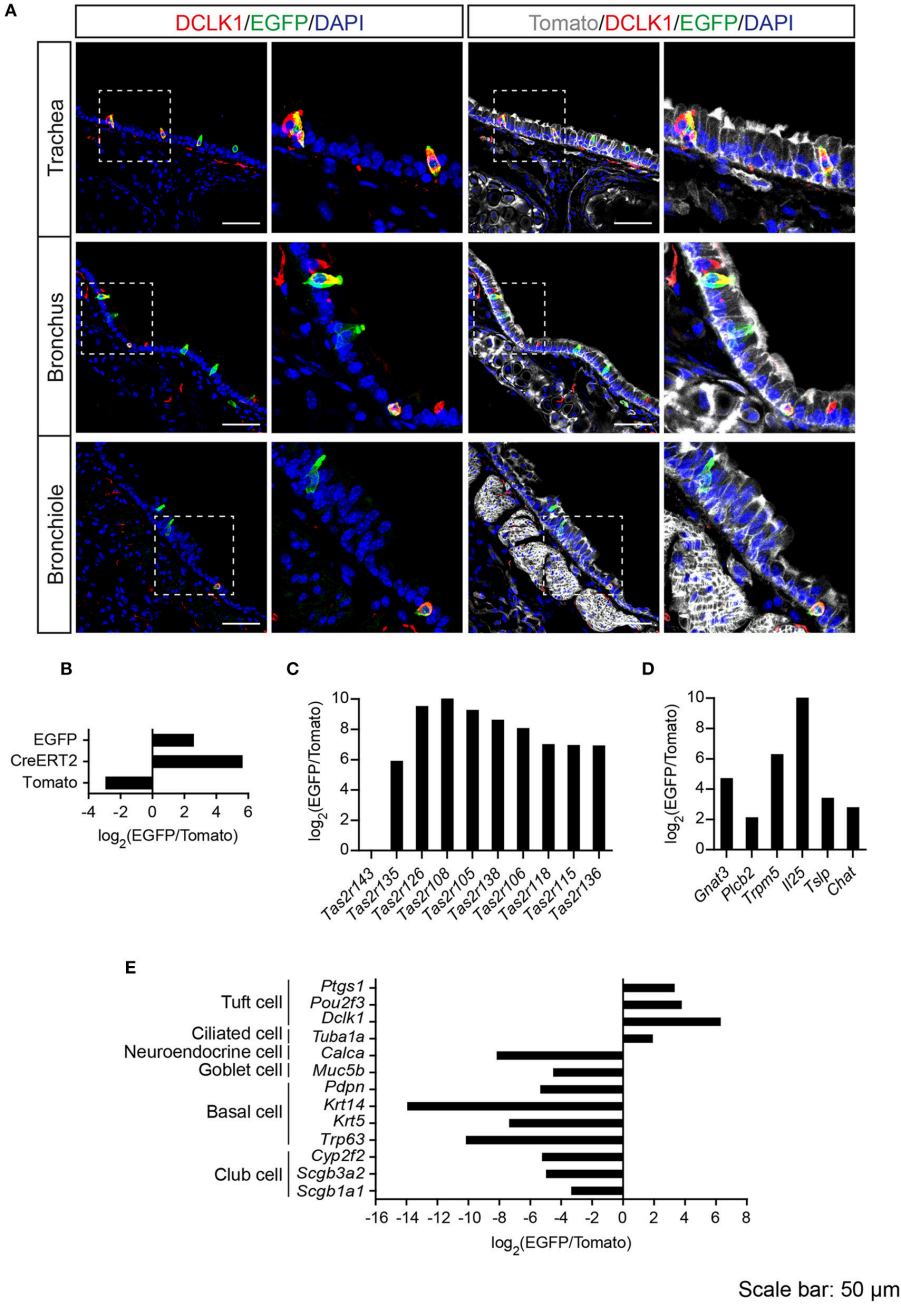
Analysis of *Tas2r143*-reporter mice in the lower respiratory tract. **(A)** Immunofluorescence staining of cryosections in the trachea, bronchus and bronchiole. EGFP-positive epithelial cells prominently colocalized with the tuft cell marker DCLK1. Nuclei were counterstained with DAPI. Squares indicate enlarged areas. Scale bars: 50 μm. **(B–E)** RNA-seq analysis of gene expression levels in purified EGFP-positive cells compared with Tomato-positive cells. The relative expression was calculated as log_2_(EGFP/Tomato). EGFP- and Tomato-positive cells were isolated from the tracheas of the reporter mice (*n* = 3).

Previous studies demonstrated solitary chemosensory cells in respiratory airways and bitter taste receptor expression in the trachea (Krasteva et al., 2011; Tizzano et al., 2011; Voigt et al., 2015). We analyzed the lower respiratory tract, which consists of the trachea, bronchi, bronchioles, and lungs. EGFP-positive cells were detected in the epithelium of trachea, primary bronchi, and rarely in the epithelium of smaller bronchi as well as bronchioles (Figure 3A; Supplementary Figure 3). The number of EGFP-positive epithelial cells in the trachea was assessed from imaging, and we found that about 4% of all epithelial cells were EGFP-positive (Table 1). The majority of EGFP-positive epithelial cells colocalized with DCLK1 (microtubule-linked protein kinase 1), which is a tuft cell marker (Gerbe et al., 2009). About 85% of tuft cells were EGFP-positive epithelial cells and about 90% of EGFP-positive epithelial cells were tuft cells (Table 2). In order to test whether EGFP-positive cells express members of the *Tas2r143*/*Tas2r135*/*Tas2r126* cluster, we isolated mRNA from EGFP-positive cells purified from the tracheas of the reporter mice by FACS (Supplementary Figure 1) and performed RNA-seq analysis. The enrichment of EGFP-positive cells was confirmed by analyzing the expression of EGFP, CreERT2 and Tomato compared with Tomato-positive cells purified from the tracheas of the reporter mice (Figure 3B). EGFP-positive cells expressed *Tas2r135, Tas2r126* and other bitter taste receptors including *Tas2r108, Tas2r105, Tas2r138, Tas2r106, Tas2r118, Tas2r115*, and *Tas2r136* (Figure 3C). In addition, EGFP-positive cells demonstrated high expression levels of genes encoding proteins in the bitter taste transduction system (*Gnat3, Plcb2, Trmp5*), acetylcholine synthesis (*Chat*; Roper, 2013) as well as cytokines responsible for type II immunity (*Il25* and *Tslp*; Gerbe and Jay, 2016; Figure 3D). Besides *Dclk1*, EGFP-positive cells highly expressed tuft cell markers *Ptgs1* and *Pou2f3* (Gerbe et al., 2016; von Moltke et al., 2016) as well as the ciliated cell marker *Tuba1a*, which is also enriched in tuft cells (Bezencon et al., 2008), but showed poor expression of marker genes for neuroendocrine cells, goblet cells, basal cells and club cells (Kotton and Morrisey, 2014; Figure 3E).

**Table 1:** Quantitative assessment of EGFP-positive epithelial cells in total epithelial cells (mean ± *SD*).

	**EGFP^+^/total epithelial cells (%)**
Trachea (*n* = 4)	3.9 ± 0.7
Stomach corpus (*n* = 4)	0.3 ± 0.1
Urethra (*n* = 3)	2.8 ± 0.7

**Table 2:** Coexpression analysis of EGFP-positive cells with tuft cell marker DCLK1 (*n* = 4, mean ± *SD*).

	**EGFP^+^DCLK1^+^/EGFP^+^ (%)**	**EGFP^+^DCLK1^+^/DCLK1^+^ (%)**
Trachea	88.5 ± 10.3	84.6 ± 2.4
Stomach corpus	100 ± 0.0	14.6 ± 4.8

### Expression of EGFP-positive cells in the gastrointestinal tract

Solitary chemosensory cells have been reported in the stomach and intestine (Chandrashekar et al., 2006; Finger and Kinnamon, 2011). Recently, these cells were shown to function as critical sentinels in the gut epithelium by promoting type II immune response (Gerbe et al., 2016; Howitt et al., 2016; von Moltke et al., 2016).

**Figure 4:**
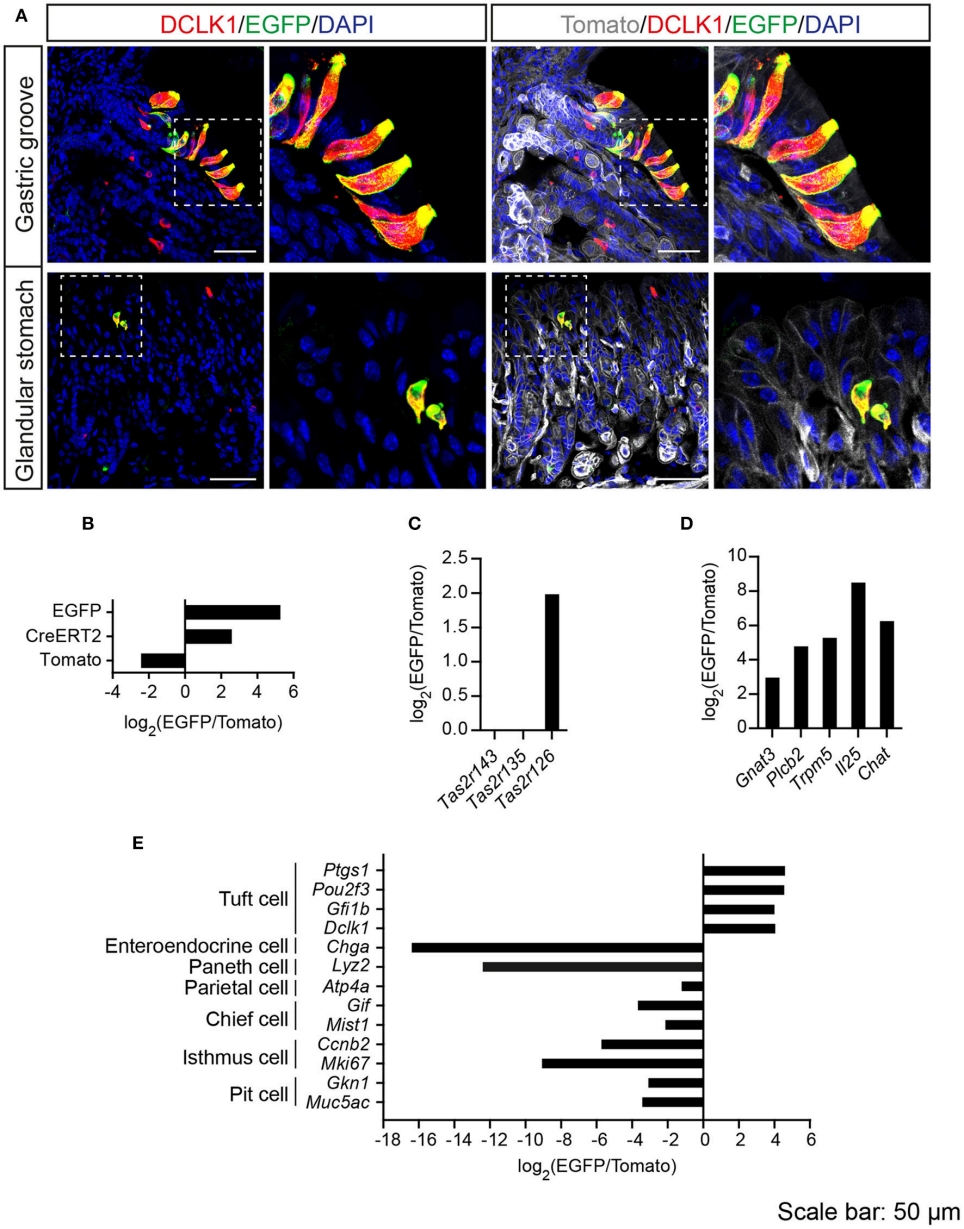
Analysis of *Tas2r143*-reporter mice in the stomach. **(A)** Immunofluorescence staining of stomach cryosections. EGFP-positive epithelial cells colocalized with the tuft cell marker DCLK1. Nuclei were counterstained with DAPI. Squares indicate enlarged areas. Scale bars: 50 μm. **(B–E)** RNA-seq analysis of gene expression levels in purified EGFP-positive cells compared with Tomato-positive cells. The relative expression was calculated as log_2_(EGFP/Tomato). EGFP- and Tomato-positive cells were isolated from the stomachs of the reporter mice (*n* = 3).

*Tas2r143*-reporter mice showed that EGFP-positive cells were clustered in the gastric groove, which is a tissue fold located at the boundary between fundus and corpus, and which is enriched by chemosensory cells (Eberle et al., 2013). EGFP-positive cells could also be detected in the epithelium of the corpus close to the gastric groove but rarely in other regions of the stomach (Supplementary Figure 3). EGFP-positive cells in both gastric groove and epithelium were to 100% colocalized with the tuft cell marker DCLK1 (Figure 4A; Table 2). In addition, EGFP-positive cells in the gastric groove were positive for α-gustducin and ChAT (choline acetyltransferase), the latter is indicating a cholinergic nature of these cells (Supplementary Figure 2). Quantitative assessment of the stomach corpus area showed that about 0.3% of total epithelial cells were EGFP-positive and about 15% of all DCLK1-positive tuft cells were EGFP-positive (Tables 1, 2). In order to test whether EGFP-positive cells express members of the bitter taste receptor cluster, we isolated mRNA from EGFP-positive cells purified from the stomachs of the reporter mice by FACS (Supplementary Figure 1) and performed RNA-seq analysis. The enrichment of EGFP-positive cells was confirmed by analyzing the expression of EGFP, CreERT2, and Tomato compared with Tomato-positive cells purified from the stomachs of the reporter mice (Figure 4B). EGFP-positive cells only expressed *Tas2r126* (Figure 4C) and demonstrated high expression levels of genes encoding proteins in the bitter taste transduction system (*Gnat3, Plcb2, Trmp5)*, acetylcholine synthesis (*Chat*; Roper, 2013) as well as the cytokine *Il25* responsible for type II immunity (Gerbe and Jay, 2016; Figure 4D). In addition to *Dclk1*, EGFP-positive cells highly expressed gastrointestinal tuft cell markers *Ptgs1, Pou2f3*, and *Gfi1b* (Gerbe et al., 2016; von Moltke et al., 2016), but showed poor expression of marker genes for enteroendocrine cells, Paneth cells, parietal cells, chief cells, isthmus cells, and pit cells (Quante and Wang, 2009; Stange et al., 2013; Figure 4E).

**Figure 5:**
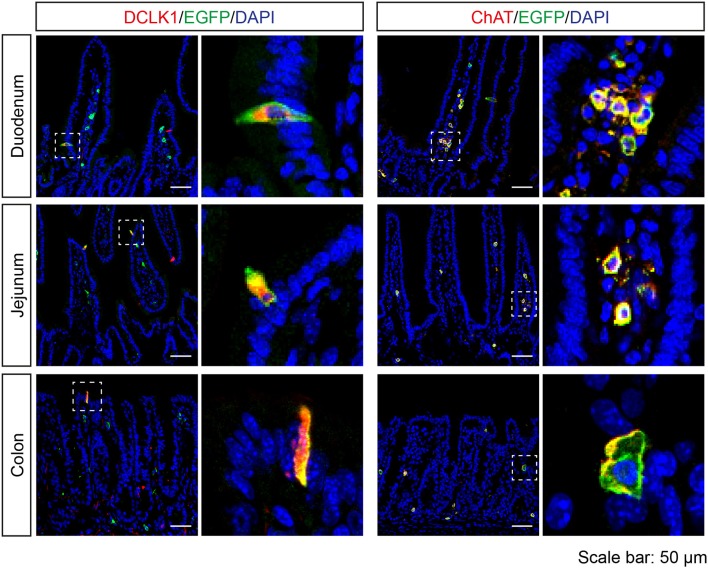
Expression analysis of *Tas2r143*-reporter mice in the intestine. Immunofluorescence staining of cryosections from the duodenum, jejunum, and colon. EGFP-positive cells were detected in the epithelium and lamina propria. EGFP-positive epithelial cells were positive for tuft cell marker DCLK1. EGFP-positive cells in the lamina propria were positive for ChAT. Nuclei were counterstained with DAPI. Squares indicate enlarged areas. Scale bars: 50 μm.

In the intestine, EGFP-positive cells were rarely found in the epithelium, but all of the EGFP-positive epithelial cells were DCLK1-positive. In addition, EGFP-positive cells were detected in the lamina propria of duodenum, jejunum, and colon, and the majority of these cells were positive for ChAT (Figure 5).

### Expression of EGFP-positive cells in the epithelium of other organs

**Figure 6:**
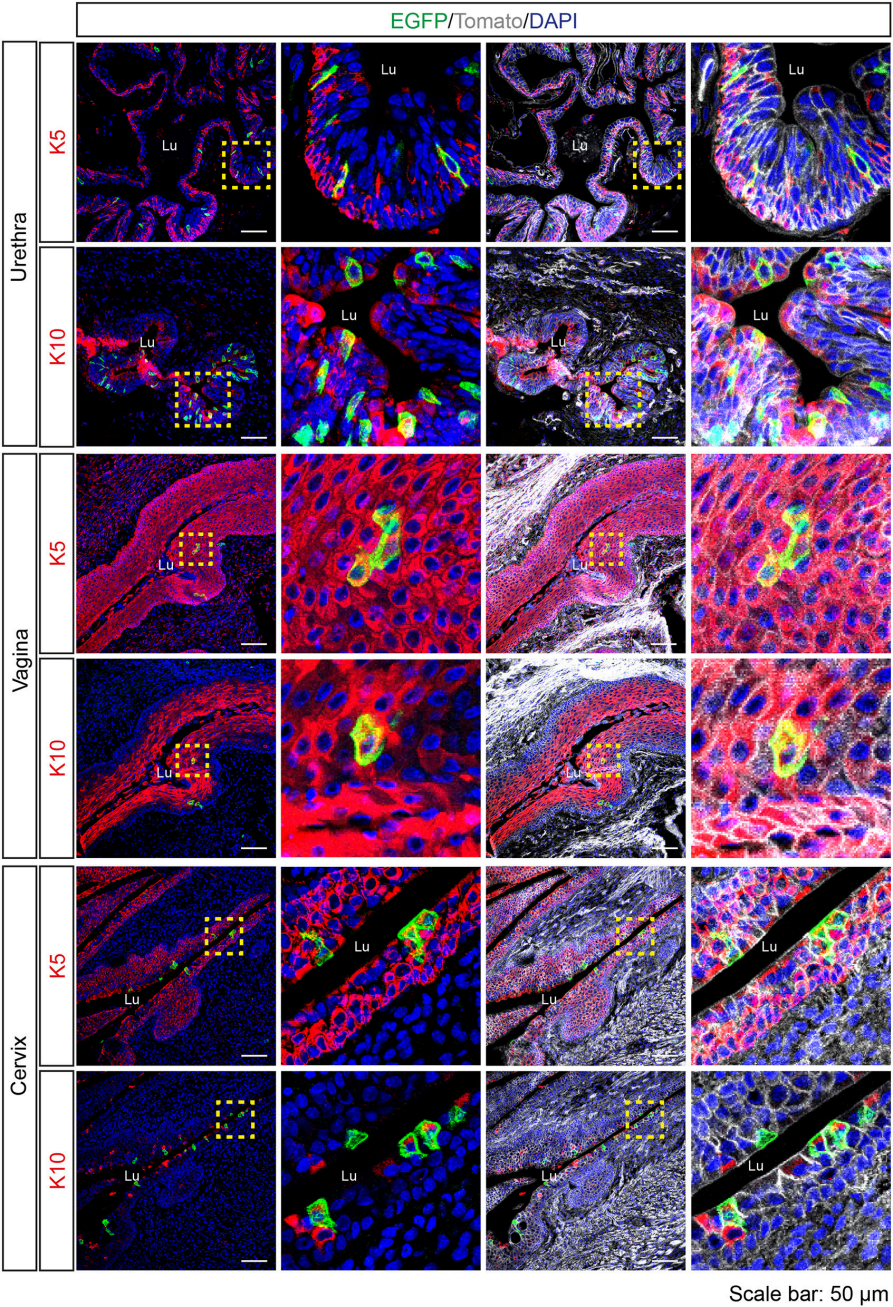
Expression analysis of *Tas2r143*-reporter mice in the urethra, vagina and cervix. Immunofluorescence staining of urethra, vagina, and cervix cryosections. EGFP-positive cells were detected in the epithelium. Mitotically active epithelial cells were stained by anti-K5 antibody. Terminally differentiated epithelial cells were stained by anti-K10 antibody. Nuclei were counterstained with DAPI. Squares indicate enlarged areas. Lu, lumen. Scale bars: 50 μm.

Since *Tas2r143*-reporter mice showed EGFP-positive cells in the epithelium of airways and the gastrointestinal tract, we investigated the epithelium in other organs and found obvious EGFP expression in the urethra, vagina and cervix. In the transitional epithelium of the urethra, EGFP-positive cells appeared to be in all layers of the epithelium and a subset of cells colocalized with mitotically active basal epithelial cell marker K5 or terminally differentiated epithelial cell marker K10. Quantitative assessment from the imaging showed that about 3% of total epithelial cells were EGFP-positive in the urethra (Table 1). In the vagina and cervix, which are lined by stratified squamous epithelium, the majority of EGFP-positive cells were found in the K5-positive mitotically active basal epithelial layer, and a small fraction of cells colocalized with terminally differentiated epithelial cell marker K10 (Figure 6).

### Expression of EGFP-positive cells in the thymus and heart

**Figure 7:**
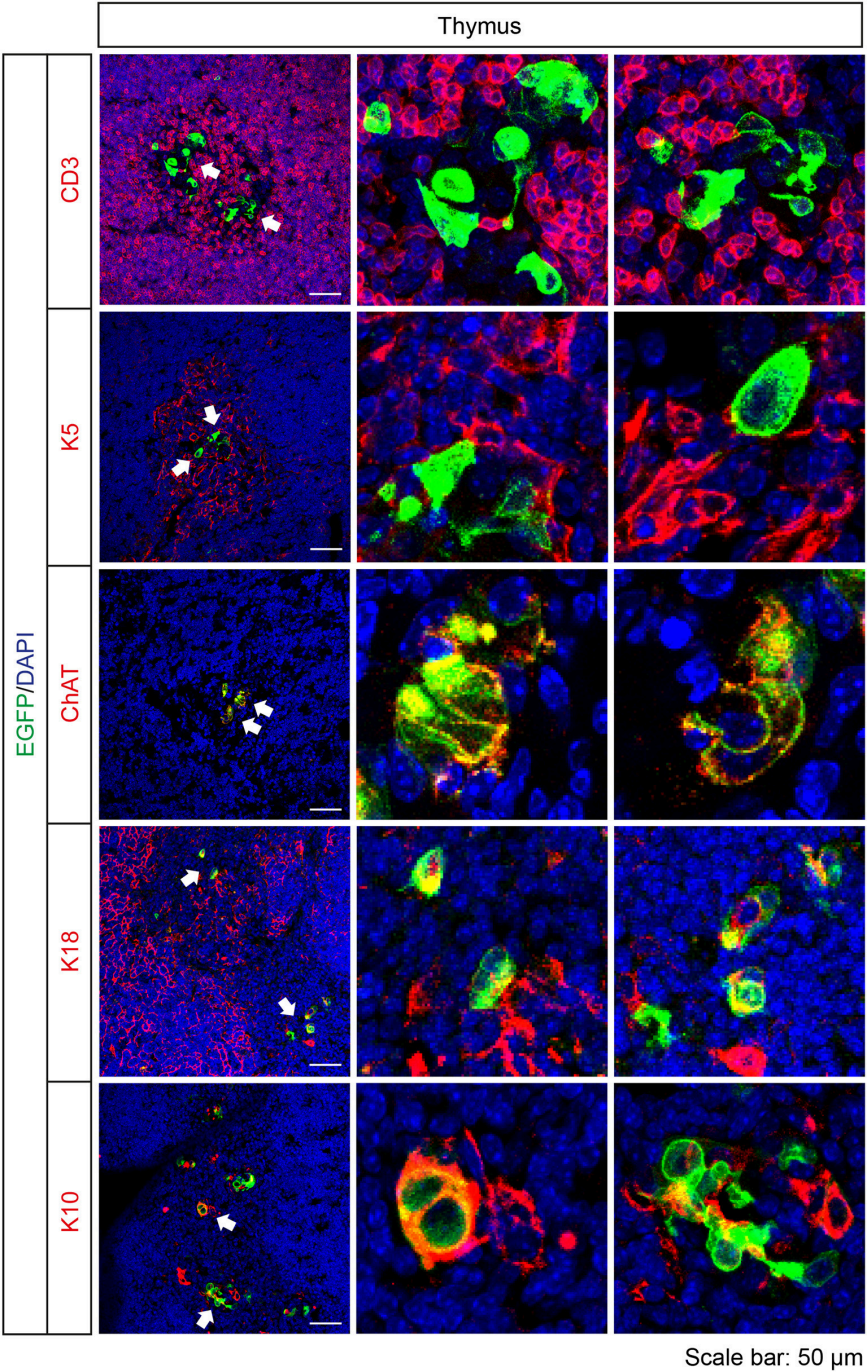
Expression analysis of *Tas2r143*-reporter mice in the thymus. Immunofluorescence staining of thymic cryosections. T cells were stained by anti-CD3 antibody. Medullar region of the thymus was marked by medullary thymic epithelial cells stained by anti-K5 antibody. Cortical region of the thymus was marked by cortical thymic epithelial cells stained by K18 antibody. EGFP-positive cells could be stained by an anti-ChAT antibody and partially colocalized with K18 and terminally differentiated epithelial cell marker K10. Nuclei were counterstained with DAPI. Arrows indicate enlarged areas. Scale bars: 50 μm.

Cortical and medullary thymic epithelial cells coordinate the development and repertoire of T cells in the thymus (Abramson and Anderson, 2017). Recently, cholinergic chemosensory cells have been reported in the thymus in ChAT- and *Tas2r131*-reporter mice (Panneck et al., 2014; Soultanova et al., 2015). Also in *Tas2r143*-reporter mice, EGFP-positive cells were detected in the thymus. These cells localized in the thymus medulla, but were negative for K5 and the T cell marker CD3. EGFP-positive cells in the thymus medulla were positive for ChAT and partially colocalized with cortical epithelial cell marker K18 and terminal differentiated epithelial cell marker K10 (Figure 7).

**Figure 8:**
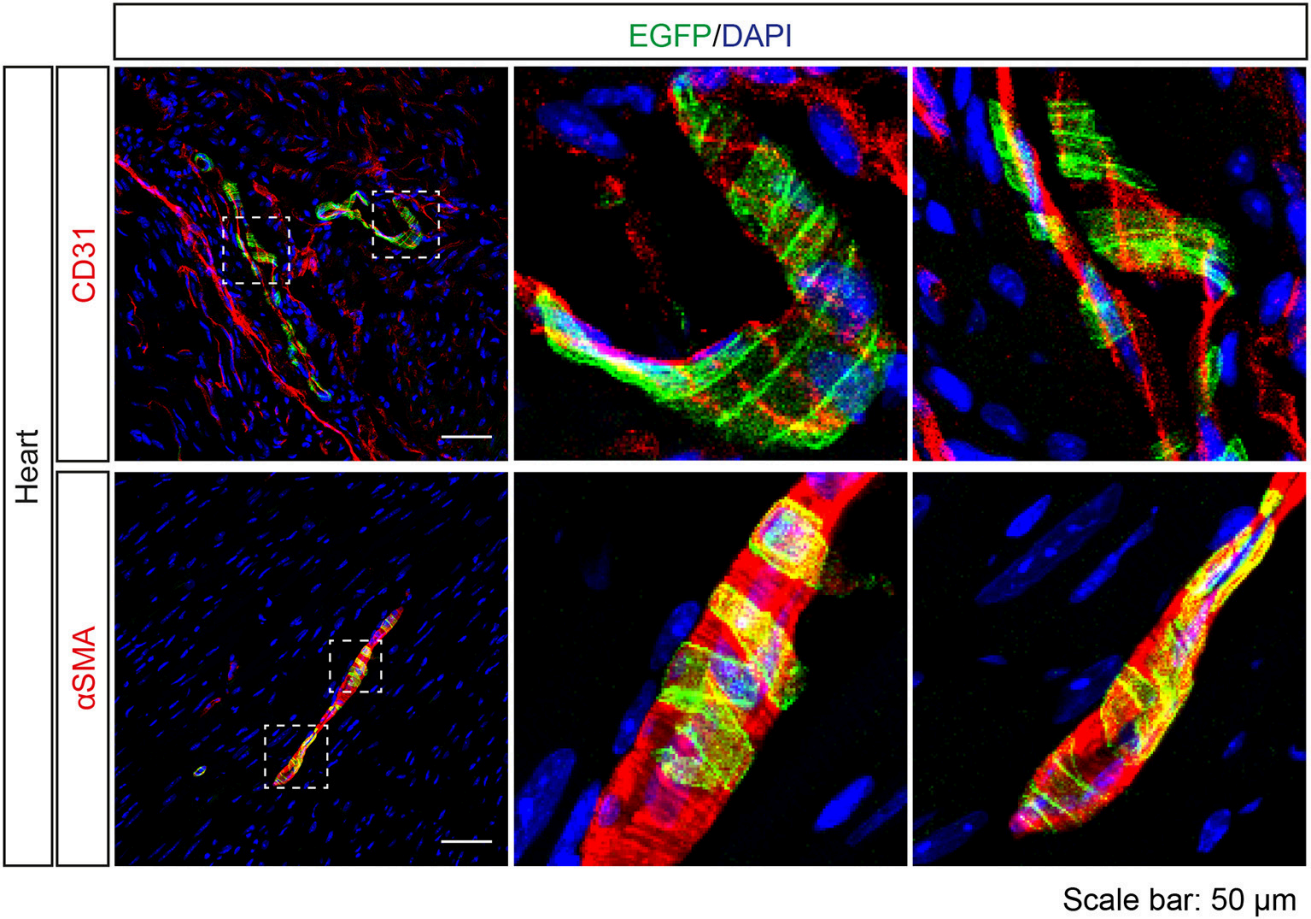
Expression analysis of *Tas2r143*-reporter mice in the heart. Immunofluorescence staining of heart cryosections. EGFP-positive cells were detected in a subset of blood vessels in the heart. EGFP-positive cells were adjacent to endothelial cells, stained by anti-CD31 antibody, and were costained with the vascular smooth muscle cell marker αSMA. Nuclei were counterstained with DAPI. Squares indicate enlarged areas. Scale bars: 50 μm.

A previous study (Foster et al., 2013) and our NanoString analysis suggested *Tas2r143/Tas2r135/Tas2r126* expression in the heart. Interestingly, EGFP-positive cells were detected in cardiac blood vessels in *Tas2r143*-reporter mice. These cells were adjacent to endothelial cells marked by CD31, and were positive for αSMA, indicating that these cells were vascular smooth muscle cells (Figure 8). Quantitative assessment from the imaging showed that 47 ± 6% (*n* = 5, mean ± *SD*) of cardiac muscularized vessels contained EGFP-positive cells.

## Discussion

The mouse bitter taste receptor genes *Tas2r143, Tas2r135*, and *Tas2r126* cluster on chromosome 6. Similarly, rat and human orthologs of these genes cluster on chromosome 4 and 7, respectively, indicating that the *Tas2r143*/*Tas2r135*/*Tas2r126* cluster is evolutionarily conserved. NanoString analysis showed expression of all three bitter taste receptors in murine hearts, which confirms previous reports indicating that *Tas2r143, Tas2r135*, and *Tas2r126* are transcribed under a common regulatory element most likely upstream of *Tas2r143* (Foster et al., 2013, 2015). Consistent with this, all cell populations, in which we detected activity of the *Tas2r143* promoter, showed expression of at least one of these receptors. RNA-seq analysis showed that EGFP-positive cells from the trachea expressed *Tas2r135*/*Tas2r126* and those from the stomach expressed *Tas2r126*, while real-time qPCR demonstrated *Tas2r143*/*Tas2r135*/*Tas2r126* expression in the tongue epithelium, which is consistent with a previous study (Lossow et al., 2016). Regulation of gene clusters has been intensively studied and expression of cluster members can be temporally and spatially regulated. For instance, members of Hox clusters are differentially expressed along the anteroposterior axis during development (Mallo and Alonso, 2013). Accordingly, it is conceivable that the divergent expression of *Tas2r143*/*Tas2r135*/*Tas2r126* in the trachea, stomach, and tongue was due to tissue-specific-transcriptional regulations. However, we cannot exclude the possibility that *Tas2r143* was expressed at very low levels in the trachea and stomach, therefore escaping the detection by RNA-seq.

EGFP-positive cells were found in organs readily exposed to pathogens, including the respiratory, gastrointestinal, and urogenital tract. Importantly, the EGFP-positive cells in the epithelium of lower airways and gastrointestinal tract were identified as tuft cells. Tuft cells were initially found in the trachea and stomach by their unique ultrastructural morphology with a “tuft” of microvilli projecting into the lumen (Jarvi and Keyrilainen, 1956; Rhodin and Dalhamn, 1956). Later they were characterized as chemosensory cells since they expressed proteins of the downstream taste signaling cascade, including α-gustducin (Hofer et al., 1996) and TRPM5 (Kaske et al., 2007). The function of tuft cells was rather unclear until three studies recently demonstrated that tuft cells initiated type II immune response by secreting the cytokine IL25, IL33, and TSLP (thymic stromal lymphopoietin) following parasite infections in the murine intestine (Gerbe et al., 2016; Howitt et al., 2016; von Moltke et al., 2016). These studies raise the hypothesis that tuft cells could directly sense microbes in the intestine through bitter taste receptors (Gerbe and Jay, 2016). Interestingly, EGFP-positive cells from our reporter mice expressed *Il25* in the trachea and stomach, indicating that members of the *Tas2r143*/*Tas2r135*/*Tas2r126* bitter taste receptor cluster may be able to sense microbial factors or other external stimuli and initiate type II immune response. In the respiratory system, activation of bitter taste receptors has been suggested to be related to innate immunity (Lee and Cohen, 2014; Lu et al., 2017). Bitter taste receptors were suggested to protect human airways against inhaled noxious irritants by increasing ciliary beating (Shah et al., 2009) or against microbes such as *P. aeruginosa* by regulating NO production, which resulted in stimulation of mucociliary clearance and direct antibacterial effects (Lee et al., 2012). In addition, bacterial quorum-sensing molecules and bitter compounds were shown to activate solitary chemosensory cells to release acetylcholine in murine airways, which triggers trigeminal reflex, leading to neurogenic inflammation responses (Saunders et al., 2014) and drop of the respiratory rate (Tizzano et al., 2010; Krasteva et al., 2012). Consistent with the role of bitter taste receptors in these cells, ChAT-positive epithelial cells were shown to express *Tas2r105* and *Tas2r108* as well as taste-signaling related proteins in the trachea, and such cells were suggested to trigger trigeminal reflex to depress the respiratory rate in response to bitter compounds (Krasteva et al., 2011). These studies suggested a protective role of bitter taste receptors in response to microbes by initiating the acetylcholine-mediated neurogenic inflammation or respiratory control to prevent further inhalation of irritants or microbes (Lu et al., 2017). Intriguingly, EGFP-positive cells from our reporter mice also expressed ChAT in the trachea and gastrointestinal system. We therefore speculate that the *Tas2r143/Tas2r135/Tas2r126* cluster can be functionally related to innate immune response in various sensory cells of the respiratory and gastrointestinal epithelium.

In the epithelium of urogenital tract, EGFP-positive cells were detected in the urethra, vagina as well as the cervix. Noteworthy, a previous study showed that chemosensory cells were restricted to the epithelium of urethra in the urinary tract (Deckmann et al., 2014), which would be consistent with our observation. EGFP-positive cells were found in the K5- and K10-positive epithelial layers. K5/K14 are major keratins of proliferative basal keratinocytes and K1/K10 are major keratins of postmitotic keratinized keratinocytes (Moll et al., 2008). Interestingly, immunostainings showed expression of TAS2R1 and TAS2R38 in the epidermis of human skin (Wolfle et al., 2015) and expression of TAS2R38 in the amniotic epithelium, syncytiotrophoblast, and decidua cells in human placenta (Wolfle et al., 2016). The bitter compounds diphenidol and amarogentin, which can activate TAS2R38 and TAS2R1, respectively, stimulated the expression of keratinocyte differentiation markers K10, involucrin, and transglutaminase 1 in human primary keratinocytes and keratinocyte cell line HaCaT, suggesting that bitter taste receptors might play a role in keratinocyte differentiation (Wolfle et al., 2015).

In the thymus, EGFP-positive cells were found in the thymic medulla, in cells positive for ChAT and partially colocalized with the cortical epithelial cell marker K18 and K10, a marker for terminal differentiated epithelial cells. These observations were similar to previous studies analyzing co-expression of ChAT and Tas2r reporter, Tas2r131 (Panneck et al., 2014; Soultanova et al., 2015). Cortical and medullary thymic epithelial cells play a critical role in the positive and negative selection of T cells, respectively (Abramson and Anderson, 2017). Thymic terminal differentiated epithelial cells are developed from medullary thymic epithelial cells, forming the Hassall's corpuscles-like structures in mice (White et al., 2010). The function of cholinergic chemosensory cells in the thymus is so far unclear.

In the phylogenetic tree of human and murine bitter taste receptor genes, *Tas2r143, Tas2r135*, and *Tas2r126* were classified into the group of one-to-one ortholog genes, which was suggested to develop from gene duplication prior to the separation of primates and rodents, suggesting a conserved function in human and mice (Shi et al., 2003). Recently, 128 naturally occurring bitter compounds were screened on murine bitter taste receptors and cognate compounds for Tas2r135 and Tasr126 have been identified (Lossow et al., 2016). Tas2r135 was responsive to 11 tested bitter compounds and was specifically activated by acesulfame K, allylisothiocyanate and salicylic acid, while Tas2r126 was responsive to 7 bitter compounds and was specifically activated by arbutin, helicon, and D-salicin (Lossow et al., 2016). These findings suggest that Tas2r135 and Tas2r126 are broadly tuned receptors (Lossow et al., 2016). It is therefore possible that members of the *Tas2r143/Tas2r135/Tas2r126* cluster are capable of sensing particular pathogens. However, it is currently unknown whether and which pathogen-derived molecules can activate Tas2r143, Tas2r135, and Tas2r126. Thus, a comprehensive screening of pathogen-derived compounds could be helpful to identify pathogen recognition pattern of these bitter taste receptors, which could be of pathophysiological relevance in mice and human.

*Tas2r143/Tas2r135/Tas2r126* and other bitter taste receptors have previously been described to be expressed in the heart based on qPCR analysis (Foster et al., 2013). To understand the function of bitter taste receptors in hearts, agonists for Tas2r143, Tas2r108, and Tas2r137 were identified and tested in Langendorff-perfused mouse hearts (Foster et al., 2014). While agonists for Tas2r108 and Tas2r137 elicited negative inotropy in the murine heart, an agonist for Tas2r143 had no effect on heart contractility (Foster et al., 2014). Surprisingly, we did not find EGFP-positive cells in cardiomyocytes but only in vascular smooth muscle cells of about 47% of cardiac muscularized vessels. We don't know the reason for the discrepancy in Tas2r143/Tas2r135/Tas2r126 expression, but we assume this might be due to a contamination of cardiomyocyte preparations by vascular smooth muscle cells. Importantly, a previous study reported TAS2R46 and Tas2r143 expression in human and murine vascular smooth muscle cells, respectively, and showed that intravenous injection of the bitter compound denatonium, which can activate TAS2R46, led to a transient drop of blood pressure in rats (Lund et al., 2013). In addition, chloroquine, denatonium, dextromethorphan, noscapine, and quinine, which are agonists for TAS2R3, TAS2R4, TAS2R10, and TAS2R14, were demonstrated to induce strong endothelium-independent relaxations in pre-contracted guinea-pig aorta (Manson et al., 2014). Dextromethorphan was demonstrated to induce vasoconstriction in pulmonary artery smooth muscle through TAS2R1 (Upadhyaya et al., 2014). In addition, bitter taste receptors were detected in human and mouse airway smooth muscle as well as in human pulmonary artery smooth muscle (Deshpande et al., 2010; Zhang et al., 2013; Camoretti-Mercado et al., 2015; Jaggupilli et al., 2017) and bitter tastants were suggested to act as potent bronchodilators (Finger and Kinnamon, 2011; Lu et al., 2017). While these studies indicate a possible role of bitter taste receptors in regulating the smooth muscle tone, the role of Tas2r143, Tas2r135, and Tas2r126 in vascular smooth muscle cells of cardiac vessels remains unclear.

In conclusion, we have established and validated the *Tas2r143*-CreERT2 transgenic mouse line. By analyzing the *Tas2r143*-CreERT2; Rosa26^flox−mT−stop−flox−mG^ reporter mouse we could observe EGFP-positive cells distributed in multiple organs. A population of EGFP-positive cells was found in the epithelium of lower airways, the gastrointestinal tract, urethra, vagina, cervix, and thymus, and EGFP-positive cells were also detected in the lamina propria of the intestine and in muscularized vessels in the heart. These data further support the concept that bitter taste receptors serve functions in the epithelium as well as the vascular system in non-gustatory tissues.

## Author contributions

SLi, NW, and SO: Conceived and designed the experiments. SLi, SLu, RX, AA, and SG: Performed the experiments and analyzed the data. SLi: Wrote the paper, with critical input mainly from NW and SO. All authors read and approved the final version of the manuscript.

### Conflict of interest statement

The authors declare that the research was conducted in the absence of any commercial or financial relationships that could be construed as a potential conflict of interest.

## Supplementary material

Supplementary Figure 1 Isolation of EGFP-positive cells by FACS Representative dot plot showing FACS analysis of EGFP-positive cells isolated from the **(A)** trachea and **(B)** stomach. Cells isolated from *Tas2r143*-reporter mice were stained with an antibody against epithelial marker EpCAM coupled to Alexa Fluor® 647. Cells isolated from Rosa26^flox−mT−stop−flox−mG^ mice were not stained and served as EGFP/EpCAM-negative control. Live single cells (blue dots) were gated by SSC/FSC and DAPI. P1 (red dots) were gated from live single cells and represented Tomato-positive cells, which were Tomato-positive/EpCAM-positive/EGFP-negative/DAPI-negative. P2 (green dots) were gated from live single cells and represented EGFP-positive cells, which are EGFP-positive/DAPI-negative.

Supplementary Figure 2 Expression of EGFP-positive cells in the gastric groove. Immunofluorescence staining of stomach cryosections. EGFP-positive cells clustered in the gastric groove and were positive for ChAT and the α-subunit of the G-protein gustducin. Nuclei were counterstained with DAPI. Squares indicate enlarged areas. Scale bars: 50 μm.

Supplementary Figure 3 Expression of EGFP-positive cells in the trachea and stomach. EGFP-positive cells were detected in the epithelium of the trachea and stomach cryosections from tamoxifen-treated reporter mice. Nuclei were counterstained with DAPI. Green fluorescent structures within the gastric cavity were debris. Scale bars: 100 μm.
