# Supplementary figures and images for: Members of Bitter Taste Receptor Cluster Tas2r143/Tas2r135/Tas2r126 Are Expressed in the Epithelium of Murine Airways and Other Non-gustatory Tissues
Source: Front Physiol. 2017 Oct 30;8:849. doi: 10.3389/fphys.2017.00849 (PMC5670347; doi:10.3389/fphys.2017.00849)

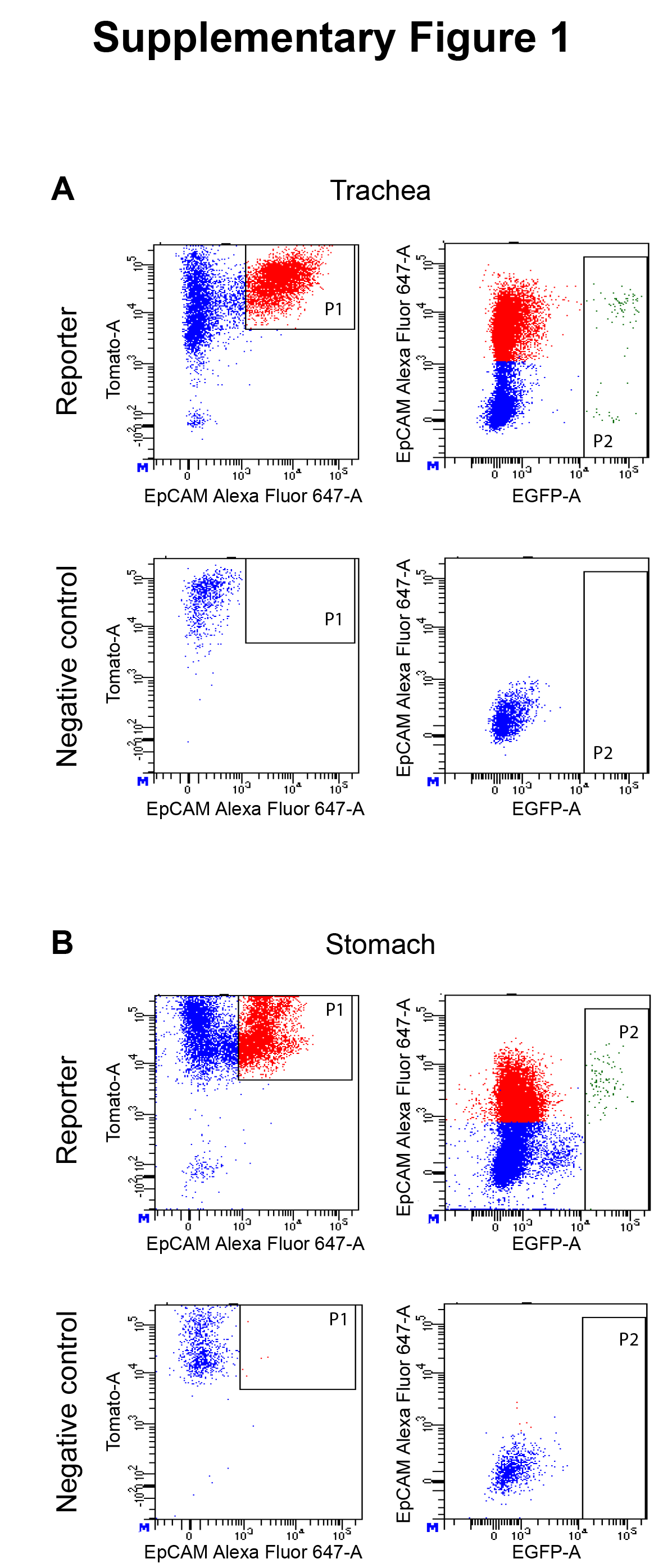

Supplement: Supplementary Figure 1 — Isolation of EGFP-positive cells by FACS Representative dot plot showing FACS analysis of EGFP-positive cells isolated from the (A) trachea and (B) stomach. Cells isolated from Tas2r143-reporter mice were stained with an antibody against epithelial marker EpCAM coupled to Alexa Fluor® 647. Cells isolated from Rosa26flox−mT−stop−flox−mG mice were not stained and served as EGFP/EpCAM-negative control. Live single cells (blue dots) were gated by SSC/FSC and DAPI. P1 (red dots) were gated from live single cells and represented Tomato-positive cells, which were Tomato-positive/EpCAM-positive/EGFP-negative/DAPI-negative. P2 (green dots) were gated from live single cells and represented EGFP-positive cells, which are EGFP-positive/DAPI-negative. [file Image1.TIF]

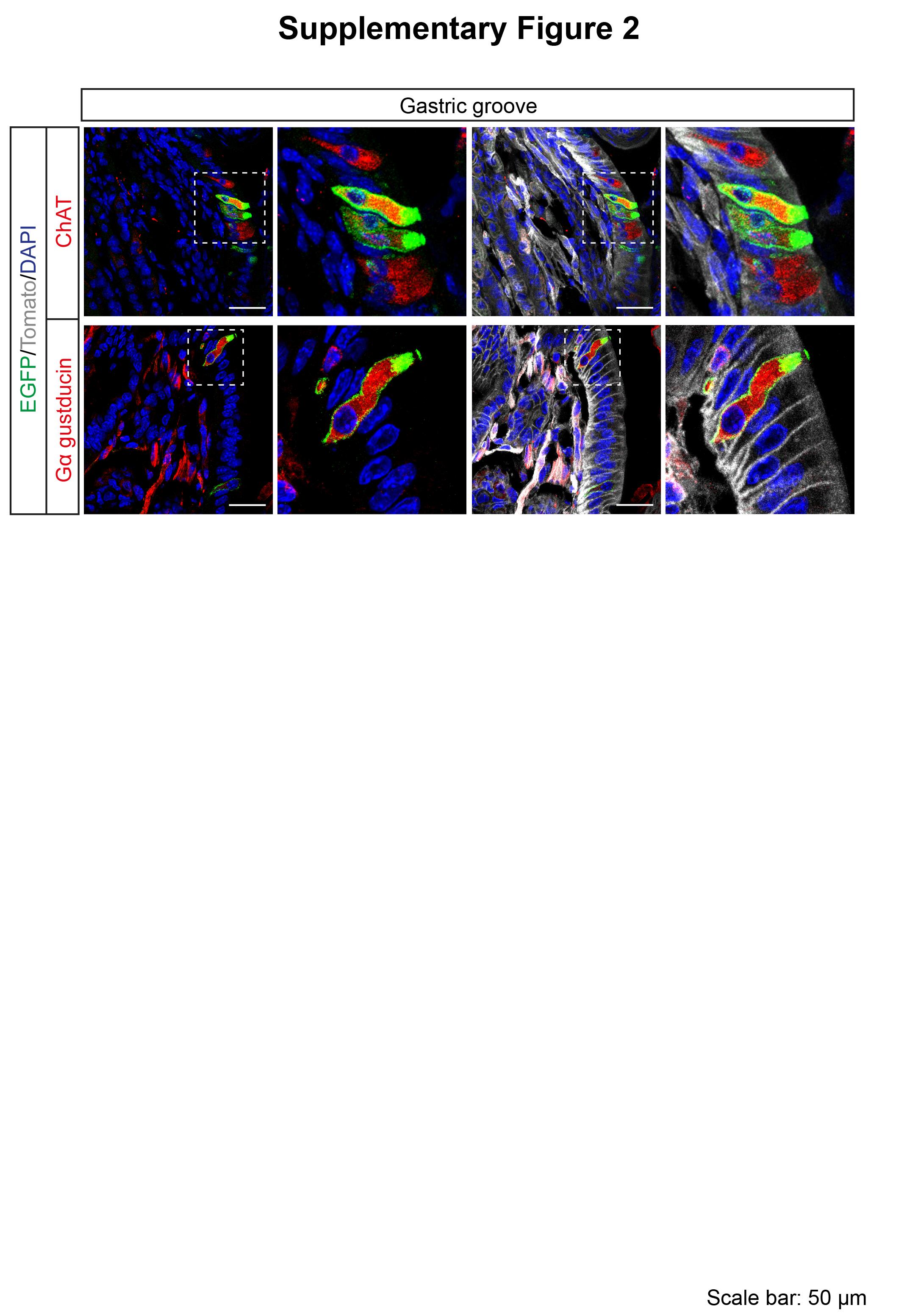

Supplement: Supplementary Figure 2 — Expression of EGFP-positive cells in the gastric groove. Immunofluorescence staining of stomach cryosections. EGFP-positive cells clustered in the gastric groove and were positive for ChAT and the α-subunit of the G-protein gustducin. Nuclei were counterstained with DAPI. Squares indicate enlarged areas. Scale bars: 50 μm. [file Image2.TIF]

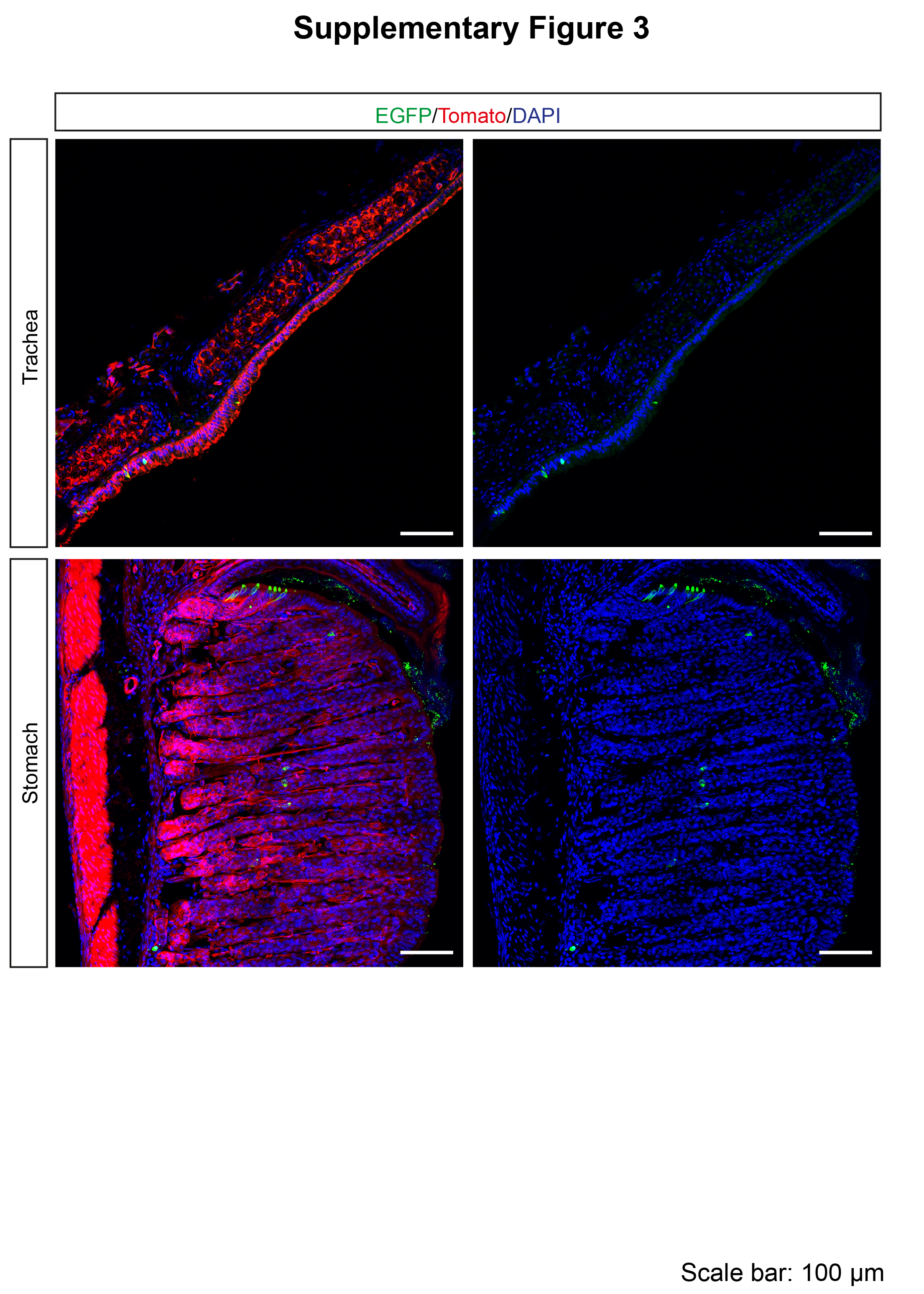

Supplement: Supplementary Figure 3 — Expression of EGFP-positive cells in the trachea and stomach. EGFP-positive cells were detected in the epithelium of the trachea and stomach cryosections from tamoxifen-treated reporter mice. Nuclei were counterstained with DAPI. Green fluorescent structures within the gastric cavity were debris. Scale bars: 100 μm. [file Image3.TIF]
